# Patient Perceptions of Artificial Intelligence-Generated Kidney Transplant Information: Comparing ChatGPT With the National Kidney Foundation

**DOI:** 10.1016/j.xkme.2026.101247

**Published:** 2026-01-08

**Authors:** Hwarang Stephen Han, Jihye Lee

**Affiliations:** 1 Division of Nephrology, Department of Internal Medicine, Dell Medical School at The University of Texas at Austin, Austin, TX; 2 Stan Richards School of Advertising and Public Relations, Moody College of Communication, The University of Texas at Austin, Austin, TX

**Keywords:** AI, generative artificial intelligence, patient education, kidney transplant

## Abstract

**Rationale & Objective:**

Generative artificial intelligence (AI) may help patients better understand the complexities of kidney transplantation. However, little is known about how individuals with chronic kidney disease (CKD) perceive AI-generated health information. This study assessed patient perceptions of AI-generated responses to common kidney transplant queries compared to those from a trusted health resource.

**Study Design:**

A cross-sectional online survey.

**Setting & Participants:**

A total of 216 adults with CKD, including kidney transplant recipients, residing in the United States participated in the study.

**Exposures:**

Participants compared kidney transplant-related query responses generated by ChatGPT (GPT-4o), a widely used generative AI tool, with those provided by the National Kidney Foundation (NKF).

**Outcomes:**

Participant perceptions across several domains: overall preference, perceived information quality, empathy, and learning outcomes.

**Analytical Approach:**

Participants reviewed paired responses from both ChatGPT and NKF, presented without source attribution. Results were analyzed using mixed-effect models.

**Results:**

Participants preferred ChatGPT-generated responses over NKF’s in 81.3% of comparisons (P < 0.001). ChatGPT responses were rated significantly higher than NKF’s in terms of information quality, empathy, and perceived learning outcomes (all *P* < 0.001).

**Limitations:**

The web-based survey may not fully represent the diverse populations served by transplant centers. Limited prompts were used, which may not capture the full range of transplant scenarios. We were also unable to determine which specific features influenced participant preferences.

**Conclusions:**

Generative AI platforms like ChatGPT may present information in ways that resonate with patients, potentially enhancing their education and engagement. However, as these tools are still in the early stages of integration into everyday life, their use should be guided by careful human oversight.

## Plain-language Summary

This study examined how adults with chronic kidney disease, including transplant recipients, engage with generative artificial intelligence (AI) to learn about kidney transplantation. Participants reviewed answers to common transplant queries from both ChatGPT, a popular AI tool, and the National Kidney Foundation, a trusted educational source, without knowing which source provided each answer. Participants preferred AI-generated responses in 81.3% of cases and rated ChatGPT higher than National Kidney Foundation in information quality, empathy, and perceived learning. These findings suggest that generative AI has the potential to present information in ways that resonate with patients. Although promising, AI tools should still be used under the guidance of healthcare professionals, as they remain in the early stages of integration into everyday life.

Generative artificial intelligence (AI) is rapidly transforming how people access information, make decisions, and navigate daily life. These AI-powered chatbots, built on large language models (LLMs) trained on large-scale text datasets and related structured data from books, websites, and other sources, can generate human-like responses to a wide range of questions and prompts.^1^ Although not specifically developed for health care, their ability to provide medical guidance is making generative AI tools increasingly valuable in clinical settings.^2^, ^3^, ^4^, ^5^, ^6^, ^7^ For example, studies have found that Chat Generative Pre-trained Transformer (ChatGPT), one of the popular generative AI services, has demonstrated the ability to pass the United States Medical Licensing Examination (USMLE) and can supply high-quality health information with a notable degree of empathy.^8^^,^^9^

The field of kidney transplantation presents a valuable opportunity for investigating the applications of generative AI in health care. Kidney transplant, a preferred treatment for patients receiving kidney replacement therapy, offers better long-term outcomes compared to dialysis.^10^ However, the transplant journey can be overwhelming for many patients, often presenting significant challenges.^11^^,^^12^ Patients tend to face complex medical decisions, undergo comprehensive pretransplant evaluations for candidacy, and continue posttransplant monitoring for potential complications for the life of the allograft.^13^ Kidney transplant centers and their multidisciplinary teams have been the primary sources of guidance, yet many patients and their support networks encounter barriers including limited time, emotional stress, geographic distance, and resource constraints.^14^^,^^15^ ChatGPT and other generative AI tools have the potential to complement traditional clinical care in the field of kidney transplantation.^16^, ^17^, ^18^, ^19^, ^20^ By delivering easily accessible, around-the-clock support and education, generative AI has the hope to enhance patient knowledge, involvement, and sense of control throughout the kidney transplant process.

Despite increasing interest in the potential of generative AI in kidney transplantation, little is known about how patients with chronic kidney disease (CKD) perceive and interpret its use in the context of kidney transplantation. This study addresses a critical gap by examining how patients with CKD perceive information provided by ChatGPT compared with that from the National Kidney Foundation (NKF), a widely trusted authority in kidney health education.^21^ From NKF’s website, we identified common kidney transplant-related queries and their official responses. Using the same queries as prompts, we generated corresponding responses from ChatGPT and asked participants to compare them with responses from NKF without knowing the source of each response. This comparative approach offers valuable insights into how generative AI is perceived in terms of quality and usefulness compared to traditional web-based information. It also reveals patient preferences, highlighting both the strengths and limitations of generative AI in kidney transplantation education, while identifying opportunities to enhance the information provided by established expert sources.

## Materials and Methods

For this study, NKF’s kidney transplant educational webpage was used to develop prompts for ChatGPT and to serve as a benchmark for comparison.^22^ Four prompts were selected from NKF’s website, reflecting common queries related to kidney transplantation: ‘About kidney transplant,’ ‘Benefits of kidney transplant,’ ‘Risks of kidney transplant,’ and ‘Who can get a kidney transplant’. We first gathered NKF’s responses to the prompts. Using the same queries, we then generated corresponding transcripts from responses from ChatGPT (GPT-4o, accessed on May 22, 2025). To eliminate any potential bias from prior user interactions, we used an alias user account and cleared the chat history before entering each prompt into ChatGPT. To emulate a typical user experience, we did not manipulate ChatGPT’s parameters, including response length, temperature (which controls the randomness of the model’s output) or top_p sampling (which limits choices to the most probable next words), as changes to these parameters could influence the content, tone, and variability of its responses.^23^ Four physicians with expertise in kidney transplantation reviewed ChatGPT’s responses to assess their accuracy and clinical appropriateness. The complete responses from both NKF and ChatGPT are provided in the Supporting Information (Table S1).

Participants were recruited through Prolific, an online research panel provider known for delivering high-quality data in terms of participant attention, comprehension, and reliability.^24^ Participants who consented to the study completed a brief survey on demographic information (eg, age, gender, education, and household income) and assessed their attitudes toward AI (eg, ‘AI will make the world a better place,’ ‘I have strong negative emotions about AI’).^25^^,^^26^ Of the 237 participants initially recruited, 21 were excluded for failing an attention check intended to confirm they had reviewed both the prompts and responses. This resulted in a final analytic sample of 216 United States adults aged 18 to 78 (M = 39.41, SD = 13.55) with CKD, including individuals with a history of kidney transplantation. As shown in Table 1, nearly half (47.69%) reported having kidney disease but not being on dialysis, while 24.5% were currently treated with dialysis and 27.8% had received a kidney transplant. The sample was 53.7% female, 44% male, and 2.31% non-binary. The most represented age groups were 18-29 (27.8%) and 30-39 (28.2%). The sample overrepresented individuals with higher household incomes, with 44.4% reporting annual earnings of $100,000 or more. Participants with higher educational attainment were also overrepresented, with 50.5% holding a graduate degree and 34.3% a 4-year college degree, a proportion substantially higher than the rates reported by the United States Renal Data System.^27^ The majority identified as White (64.8%), followed by Black or African American (27.8%). Participants were geographically diverse, residing in 38 states, with the highest representation from California (10.7%), Texas (10.2%), and New York (8.8%). To address these demographic imbalances, we included demographic factors as control variables in the analysis. The model included key covariates such as age, gender, race, education, income, kidney disease status, and attitudes toward AI. This approach helps ensure that estimates of the main effect of system are not confounded by demographic differences in the sample.

**Table 1:** Study Participant Characteristics (N =216)

Characteristic	Category	Count (%)
Type of Kidney disease	Has kidney disease but not treated with dialysis	103 (47.7%)
	Currently treated with dialysis	53 (24.5%)
	Has received a kidney transplant	60 (27.8%)
Age	18-29	60 (27.8%)
	30-39	61 (28.2%)
	40-49	44 (20.4%)
	50-59	29 (13.4%)
	60+	22 (10.2%)
Gender	Female	116 (53.7%)
	Male	95 (44%)
	Non-binary	5 (2.3%)
Education	High school diploma or less	10 (4.6%)
	Some college, no degree	12 (5.6%)
	2-year college degree	11 (5.1%)
	4-year college degree	74 (34.3%)
	Graduate degree (Master's, PhD, MD, etc.)	109 (50.5%)
Household income	Less than $50,000	43 (19.9%)
	$50,000-$99,999	77 (35.6%)
	$100,000 or more	96 (44.4%)
Race/ethnicity	American Indian or Alaska Native	2 (0.9%)
	Asian or Asian-American	5 (2.3%)
	Black or African-American	60 (27.8%)
	Hispanic or Latino or Spanish Origin	5 (2.3%)
	Some other race	4 (1.9%)
	White	140 (64.8%)
Number of observations		216 (100%)

**Figure 1:**
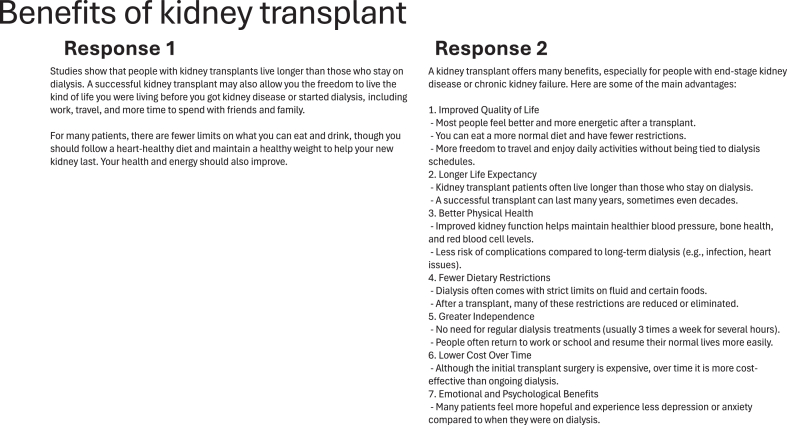
Sample screenshot showing a transcript of a kidney transplant query. Response 1 represents the answer provided by the National Kidney Foundation (NKF), while Response 2 shows the corresponding response generated by ChatGPT.

Each participant was shown transcripts containing prompts paired with responses from ChatGPT or original content from NKF, presented in random order without revealing the source. NKF and ChatGPT responses were randomized per participant and per prompt to minimize potential ordering effects. To ensure data quality, participants were required to spend at least 30 seconds reading each transcript. For each transcript, participants evaluated responses across 3 dimensions: information quality, empathy, and perceived learning outcomes. Information quality (eg, “How would you rate the quality of the information provided?”) and empathy (eg, “How empathetic do you find the information provided?”) were assessed using 5-point Likert scales (1 = very low and 5 = very high), with higher values indicating greater information quality or empathy.^8^ Perceived learning outcomes were measured by participants’ agreement with two statements:^28^ “The response I just read helped me better understand kidney transplantation,” and “After reading the response, I felt more confident in my understanding of kidney transplantation.” These were also rated on 5-point Likert scales (1 = strongly disagree and 5 = strongly agree). Internal consistency for the two efficacy items was assessed using Pearson correlations for each response, which indicate how closely the items are related to each other. Correlations ranged from 0.69 to 0.82 across NKF and ChatGPT responses, indicating acceptable reliability for composite efficacy scores. Thus, 2 items were averaged to create a single composite score for perceived learning outcomes, with higher values indicating greater perceived learning. Finally, after reading 2 responses to the same prompt, participants were shown both responses side by side (Example shown in Fig 1) and asked to indicate which one they preferred (“Which response was better?”).^8^ Each participant reviewed a total of 8 transcripts, corresponding to 4 prompt-response pairs.

We assessed the readability of ChatGPT’s responses compared with NKF materials, using the response length (measured in word count) and the Flesch–Kincaid grade-level metric. Next, we conducted χ^2^ tests to examine whether participants’ response preferences differed between NKF and ChatGPT across the 4 prompts. We then explored how these preferences related to participants’ perceptions of information quality, empathy, and learning outcomes by comparing ratings between NKF and ChatGPT. Because each participant evaluated 8 transcripts (4 prompt-response pairs), each participant contributed multiple ratings, resulting in repeated measurements. To account for this structure, we employed mixed-effects models, which adjusts for differences between individuals and prompts. The model also controlled for participants’ demographics (age, gender, race/ethnicity, education, and household income), general attitudes toward AI, and type of CKD. All statistical analyses were performed in R statistical software, version 4.1 (R Project for Statistical Computing). This study was approved by the institutional review board at the corresponding author’s university, and all data was collected in accordance with the approved research protocol.

## Results

We began by assessing the accessibility and length of responses from NKF and ChatGPT. ChatGPT responses were longer (M = 219.0, SD = 120.87, min = 94, max = 345) compared with NKF materials (M = 164.8, SD = 18.62, min = 197, max = 241), but this difference did not reach statistical significance (V = 7, *P* = 0.63). The analysis of the Flesch–Kincaid grade-level metric, where higher scores indicate more difficult text, showed that ChatGPT responses had a higher average reading level (M = 17.12, SD = 6.83) than NKF responses (M = 9.85, SD = 1.30). However, a Wilcoxon signed-rank exact test indicated that this difference was not statistically significant (V = 9, *P* = 0.25).

Across all 864 comparisons (216 participants × 4 prompts), ChatGPT’s response was preferred in 81.3% of cases (95% CI, 78.7%-83.9%). A χ^2^ test confirmed that this overall preference for ChatGPT over NKF was statistically significant (χ^2^ = 337.5; *P* < 0.001). Each prompt, along with participants’ preferences between NKF and ChatGPT responses, is presented in Table 2.

**Table 2:** Distribution of Participant Preferences across 4 Kidney Transplantation Prompts

	Q1: About kidney transplant	Q2: Benefits of kidney transplant	Q3: Risks of kidney transplant	Q4: Who can get a kidney transplant	Total
NKF	74 (34.3%)	25 (11.6%)	31 (14.4%)	32 (14.8%)	162 (18.8%)
ChatGPT	142 (65.7%)	191 (88.4%)	185 (85.7%)	184 (85.2%)	702 (81.3%)
Total	216 (100%)	216 (100%)	216 (100%)	216 (100%)	864 (100%)

Table 3 presents the full results of the mixed-effects analyses for patient perceptions of information quality, empathy, and learning outcomes by source. In all cases, models controlled for patient demographics (eg, education and income), personal experience with kidney disease, and attitudes toward AI. For information quality, patients with CKD perceived ChatGPT’s responses as significantly higher in quality than NKF’s responses (b = 0.63, SE = 0.03; *P* < 0.001). The mean rating for ChatGPT’s responses was 4.33 (SD = 0.80, 95% CI [4.28-4.39]), whereas NKF’s responses received a lower average rating of 3.7 (SD = 0.98, 95% CI [3.64-3.77]), reflecting a 14.6% lower score on average compared with ChatGPT.

**Table 3:** Effects of Source (ChatGPT vs National Kidney Foundation) on Perceived Information Quality, Empathy, and Learning Outcomes Among Patients with Chronic Kidney Disease: Mixed-Effect Analysis Results

	Information Quality		Empathy		Learning Outcomes	
	B	SE	B	SE	B	SE
**Fixed effects**						
Intercept	2.92^a^	0.46	2.59^a^	0.57	3.00^c^	0.44
Source: ChatGPT (vs NKF)	0.63^a^	0.03	0.31^a^	0.04	0.6^a^	0.03
Control variables						
Age	0.002	0.003	−0.002	0.004	−0.001	0.003
Gender (Ref. Non-binary)						
Female	0.04	0.08	0.04	0.10	0.07	0.08
Male	0.25	0.26	−0.07	0.33	0.15	0.26
Race/Ethnicity (Ref. Other)						
American Indian or Alaska Native	−0.17	0.49	0.53	0.62	0.09	0.47
Asian	−0.9^b^	0.39	−0.47	0.49	–0.55	0.38
Black or African American	−0.43	0.3	−0.09	0.37	–0.45	0.29
Hispanic, Latino, or Spanish Origin	−0.03	0.38	0.33	0.48	−0.28	0.37
White	−0.41	0.29	0.001	0.37	−0.4	0.28
Education						
High School degree or less	0.17	0.2	0.28	0.25	0.15	0.19
Some college (no degree)	0.16	0.18	0.23	0.22	0.12	0.17
Associate degree	−0.003	0.2	0.25	0.25	0.02	0.19
Bachelor’s degree	0.01	0.09	−0.12	0.11	−0.06	0.09
Income (Ref. $300,000 or more)						
Less than $50,000	−0.1	0.21	−0.29	0.27	−0.05	0.21
$50K-$99,999	0.13	0.2	−0.2	0.25	0.15	0.2
$100k-$200,999	0.05	0.2	−0.23	0.25	0.07	0.2
AI Attitudes	0.22^c^	0.05	0.25^c^	0.07	0.23^c^	0.05
Kidney Disease (Ref. Has kidney disease but not on dialysis)						
Currently on dialysis	0.18	0.10	0.32∗	0.13	0.15	0.1
Has received kidney transplant	0.25^b^	0.1	0.38^c^	0.12	0.18	0.1
**Random Effects**						
VAR (Intercept Participants)	0.25		0.41		0.24	
VAR (Intercept Prompts)	0.03		0.001		0.01	
Residual	0.49		0.67		0.45	
**Model Fit Indices**						
Marginal R^2^	0.17		0.09		0.17	
Conditional R^2^	0.47		0.44		0.46	
AIC	4,117.09		4,656.59		4,073	
BIC	4,242.55		4,782.05		4,199.09	
Number of Observations	1,728					

*Notes:* Cell entries are mixed-effects model coefficients, when controlling for participants’ demographics (age, gender, race/ethnicity, education, and household income), attitudes toward AI, and types of kidney diseases. The number of observations comprises responses from 216 participants who evaluated transcripts across 4 distinct prompts from 2 sources (NKF vs ChatGPT; 1,728 = 216 × 4 × 2).

Abbreviations: AIC, Akaike information criterion; BIC, Bayesian information criterion; B, estimates; Conditional R^2^ (variance explained by fixed and random effects); Marginal R^2^ (variance explained by fixed effects); Ref, reference category; SE, standard error; VAR, variance.

a *P <* 0.01.

b *P <* 0.05.

c *P <* 0.001.

Table 3 further shows that ChatGPT’s responses received significantly higher empathy ratings compared to NKF (b = 0.31, SE = 0.04; *P* < 0.001). The mean empathy score for ChatGPT’s responses was 3.77 (SD = 1.08, 95% CI [3.7-3.85]), compared with 3.46 for NKF responses (SD = 1.03, 95% CI [3.39-3.53]), an 8.2% lower score on average compared with ChatGPT.

In terms of learning outcomes, participants once again rated ChatGPT’s responses more favorably than those from NKF (see Table 3). ChatGPT’s responses were perceived as significantly more educational than NKF’s responses (b = 0.6, SE = 0.03; *P* < 0.001). On average, participants gave ChatGPT responses a learning score of 4.29 (SD = 0.75, 95% CI [4.24-4.34]). In contrast, NKF responses received an average rating of 3.7 (SD = 0.95, 95% CI [3.63-3.76]), a 13.8% lower score in perceived educational value.

## Discussion

Overall, our findings demonstrate a consistent and statistically significant patient preference for ChatGPT-generated responses over content from NKF. A substantial majority of participants (81.3%) favored ChatGPT’s responses. Participants rated ChatGPT’s responses higher in information quality and empathy and reported greater self-perceived learning gains compared with NKF materials. Exploratory analyses of the responses’ readability and length showed that ChatGPT responses were longer and had higher readability level than NKF materials, although these differences were not statistically significant. These results suggest that generative AI may present information in a way that better meets patient needs and expectations, potentially filling gaps left by traditional educational resources.

The findings presented should be considered within the context of several limitations. Our study relied on web-based panel recruitment, which may not fully represent the diverse populations served by various transplant centers. The selected cohort in this study had, on average, higher levels of income and education, which may be associated with greater literacy and a preference for more detailed information that ChatGPT provided. ChatGPT responses tended to be longer and more consistent in length (i.e., lower standard deviation) than NKF materials. Although this difference was not statistically significant, likely due to the small number of observations (4 pairs per source), longer responses may have offered the deeper explanations participants preferred and conveyed a greater sense of empathy. ChatGPT outputs were also written at a higher grade level, but this difference was similarly not statistically significant. NKF materials appear to be designed for accessibility, as reflected by their Flesch–Kincaid grade-level score (M = 9.85, corresponding to a 9th-10th grade reading level). Their shorter responses may also be better suited for patients with CKD, who often have lower health literacy. Although we attempted to account for demographic imbalances by including education, income, and other variables as controls, representativeness remains limited. Nevertheless, including participants from 38 US states provides valuable insights into perceptions of ChatGPT in the kidney transplant process, and future research should validate these findings in more diverse populations.

ChatGPT’s responses were also evaluated using only four prompts from NKF. This restriction was intended to ensure consistency and focus, allowing participants to provide more reliable and uniform assessments. However, the limited number of prompts may not adequately reflect the broad spectrum of scenarios encountered in kidney transplantation. Additionally, we were unable to examine the underlying factors driving these preferences, such as language features (eg, use of emojis), emotional tone, or interface navigability. Future research should clarify these distinctions.

We selected NKF as a reference point because it represents the type of educational material patients commonly access online, providing a clinically meaningful basis for comparing AI-generated content with resources patients are already likely to use. Although NKF materials are developed and reviewed by clinical experts, they are written for broad patient education rather than as targeted expert-level responses. As such, they do not replace a true human-expert comparator for the specific prompts used in this study.

Most research on generative AI in healthcare has primarily focused on clinical expert evaluations of its accuracy and utility.^8^^,^^19^^,^^20^^,^^29^, ^30^, ^31^, ^32^, ^33^, ^34^ Although expert validation of generative AI in health care is essential, understanding patients’ perspectives is equally important, as they have a deeply personal stake in their care and often have informational needs that differ significantly from those of clinicians. One study examining patient perceptions of generative AI responses to common health questions found that ChatGPT was perceived as more empathetic and useful than physicians responding on web-based forums.^35^ However, patient’s perceptions of generative AI use remain underexplored in the field of kidney transplantation. Another study suggests that the perceptions of ChatGPT’s information quality in kidney transplantation vary according to general users’ racial/ethnic and educational backgrounds, highlighting potential disparities and important considerations for implementing generative AI tools in health care education.^36^ This highlights the urgent need to understand how patients with CKD perceive AI-generated information related to kidney transplantation.

Each new version of ChatGPT has demonstrated better performance in answering kidney transplant related questions, suggesting continued improvement is likely.^37^ Although numerous generative AI tools are available, this study utilized ChatGPT because of its widespread popularity.^38^ Although newer models may offer greater accuracy and enhanced safety, survey responses were not re-collected using these versions. Exploratory analyses comparing GPT-4o and GPT-5.1 (accessed on November 15, 2025) responses in terms of response length and Flesch–Kincaid grade level suggested that the outputs between the GPT versions are generally comparable (see Table S2 in the Supporting Information). These findings support the utility of our study as a baseline and provide a foundation for future research evaluating newer versions (eg, GPT-5.2) of generative AI in kidney transplant. Future research should also investigate the use of alternative models (eg, Gemini, Meta AI, Claude, and Grok), and generative AI specifically designed for health care or transplantation. Different generative AI models may yield varied results due to differences in their training methods. Examining how health information is perceived when delivered by accessible, general-purpose generative AI models versus specialized platforms could offer valuable insights into the effectiveness and suitability of these tools for communicating medical knowledge.

Although not fully explored in this study, generative AI offers the advantage of a conversational format that encourages follow-up questions and fosters interactive engagement, which static webpages are less equipped to provide. In addition, because generative AI is trained on vast and diverse datasets extending far beyond a single website, their results may be broader and less constrained by the perspective of any single source. Educational website organizers may therefore consider incorporating generative AI tools to utilize these benefits. Furthermore, generative AI holds great potential for non-English speakers, as it can function as a real-time translator, enabling users to access information across language boundaries.^39^^,^^40^ Future research should explore full back-and-forth interactions between users and generative AI to gain deeper insight into patients’ needs and to better define the role of generative AI in health care education and communication.

Generative AI remains in its early stages of integrating into people’s daily lives, and its adoption in health care must proceed with caution.^41^ ChatGPT may outperform traditional web searches in aiding preliminary diagnosis but has potential for misinformation and confusion.^42^ It is important to note that generative AI can occasionally produce responses that are grammatically correct and seemingly plausible but factually inaccurate (hallucinations), which could misinform users if left unchecked.^43^ Although our expert review found no major inaccuracies in ChatGPT’s responses in this study, the limited scope of prompts prevents a comprehensive assessment of its accuracy across the full range of kidney transplant-related queries. Past studies have suggested that using ChatGPT to answer clinical questions can sometimes lead to inaccurate responses with the potential for harm, underscoring the need for human oversight.^20^^,^^33^ Therefore, we recommend using generative AI as a complementary tool to traditional educational resources and human expertise, rather than as a standalone solution.

Our study represents an initial step toward understanding how generative AI may enhance patient education and engagement in the context of kidney transplantation. Although transplant centers and their multidisciplinary teams should remain the primary source of most trusted information, generative AI can serve as a valuable supplement. By combining the accessibility and scalability of generative AI with expert human oversight, transplant teams may be able to deliver more engaging, empathetic, and effective informational support to patients navigating complex treatment decisions.
