# Supplementary material for: Patient Perceptions of Artificial Intelligence-Generated Kidney Transplant Information: Comparing ChatGPT With the National Kidney Foundation
Source: Kidney Med. 2026 Jan 8;8(3):101247. doi: 10.1016/j.xkme.2026.101247 (PMC12936935; doi:10.1016/j.xkme.2026.101247)
Supplement: Supplementary File (PDF) — Tables S1-S2. [file mmc1.pdf]

Table S1. Prompts with Responses from the National Kidney Foundation (NKF) and ChatGPT

**Prompt**	**NKF**	**ChatGPT**
1. About Kidney Transplant	When your kidneys fail, treatment is needed to replace the work your own kidneys can no longer do. There are two types of treatment for kidney failure: dialysis or transplant.<br><br>When you get a kidney transplant, a healthy kidney is placed inside your body to do the work your own kidneys can no longer do. On average, a kidney transplant from a living donor lasts about 15 to 20 years, and a kidney from a deceased donor lasts 8 to 12 years. Some will last longer; others will last less.<br><br>Today, most people get a kidney transplant after being on dialysis for some amount of time, but that is not the only option. Getting a transplant before you need to start dialysis is called a preemptive transplant. Research shows that a preemptive or early transplant, with little or no time spent on dialysis, can lead to better long-term health. It may also allow you to keep working and have a better quality of life.<br><br>If your doctor has told you that your kidney disease is getting worse or that you are in a later stage of kidney disease, called Stage 3B, Stage 4,	What is a Kidney Transplant?<br>A kidney transplant is a surgical procedure where a healthy kidney from a living or deceased donor is placed into someone whose kidneys no longer function properly (usually due to end-stage kidney disease).<br><br>How Does It Work?<br>The new kidney is placed in the lower abdomen.<br>It usually starts working immediately or within a few days.<br>The old (non-functioning) kidneys are often left in place, unless there's infection or other complications.<br><br>Who Needs It?<br>People with:<br>Chronic kidney disease (CKD)<br>End-stage renal disease (ESRD)<br>Kidney failure that can't be managed with dialysis long-term<br><br>Eligibility Criteria<br>Overall good health (aside from kidney issues)<br>No active infections or cancers<br>Willingness to take immunosuppressive drugs for life

Prompt	NKF	ChatGPT
	or Stage 5, it could be a good time to talk about kidney transplant.<br><br>Types: Living and Deceased<br><br>There are two types of kidney transplants: a deceased donor kidney transplant and a living donor kidney transplant. A deceased donor kidney comes from someone has died and made the decision to become an organ donor. A kidney from a living donor is from someone who is living, has two healthy kidneys, and chooses to donate one to someone in need. A living donor can be someone you know, like a family member or friend, but they do not need to be related to you.<br><br>The first step to getting a deceased or living donor kidney is to have an evaluation at a transplant center. In most cases, a potential living donor can be evaluated at the same time as the recipient, who is the person with kidney disease who needs a kidney.	Strong support system and ability to attend follow-ups<br><br>Types of Donors<br>Living Donor: A relative, friend, or even altruistic stranger. Living donations often last longer and work better.<br>Deceased Donor: Someone who has died, usually from brain death, and donated their organs.<br>Benefits Over Dialysis<br>Better quality of life<br>Fewer dietary restrictions<br>Longer life expectancy<br>Freedom from dialysis routines<br><br>Risks and Considerations<br>Rejection of the organ<br>Side effects of immunosuppressants (infections, higher cancer risk)<br>Surgical risks (bleeding, clots)<br>Need for lifelong follow-up<br><br>🛠Recovery and Aftercare<br>Hospital stay: ~1 week<br>Recovery time: 6–8 weeks<br>Medications for life: Anti-rejection drugs (immunosuppressants) to prevent the body from attacking the new kidney<br>Regular blood tests to monitor function

Prompt	NKF	ChatGPT
2. Benefits of kidney transplant	Studies show that people with kidney transplants live longer than those who stay on dialysis. A successful kidney transplant may also allow you the freedom to live the kind of life you were living before you got kidney disease or started dialysis, including work, travel, and more time to spend with friends and family.<br><br>For many patients, there are fewer limits on what you can eat and drink, though you should follow a heart-healthy diet and maintain a healthy weight to help your new kidney last. Your health and energy should also improve.	A kidney transplant offers many benefits, especially for people with end-stage kidney disease or chronic kidney failure. Here are some of the main advantages:<br><br>1. Improved Quality of Life<br>Most people feel better and more energetic after a transplant.<br>You can eat a more normal diet and have fewer restrictions.<br>More freedom to travel and enjoy daily activities without being tied to dialysis schedules.<br><br>2. Longer Life Expectancy<br>Kidney transplant patients often live longer than those who stay on dialysis.<br>A successful transplant can last many years, sometimes even decades.<br><br>3. Better Physical Health<br>Improved kidney function helps maintain healthier blood pressure, bone health, and red blood cell levels.<br>Less risk of complications compared to long-term dialysis (e.g., infection, heart issues).<br><br>4. Fewer Dietary Restrictions<br>Dialysis often comes with strict limits on fluid and certain foods.

Prompt	NKF	ChatGPT
		After a transplant, many of these restrictions are reduced or eliminated.<br><br>5. Greater Independence<br>No need for regular dialysis treatments (usually 3 times a week for several hours).<br>People often return to work or school and resume their normal lives more easily.<br><br>6. Lower Cost Over Time<br>Although the initial transplant surgery is expensive, over time it is more cost-effective than ongoing dialysis.<br><br>7. Emotional and Psychological Benefits<br>Many patients feel more hopeful and experience less depression or anxiety compared to when they were on dialysis.
3. Risks of kidney transplant	Having a kidney transplant does not "cure" kidney disease. There are also risks, including the risks of surgery. After the transplant, you will need to take anti-rejection medicines, also called immunosuppressants, for as long as your new kidney is working, which can have side effects. You will have a higher risk for infections and certain types of cancer.<br><br>Although most transplants are successful and last for many years, how long they last can vary from one person to the next. Depending on your age,	Medical Risks<br>Rejection of the Kidney<br>Your immune system may see the new kidney as a foreign object and try to attack it.<br>Even with immunosuppressive medications, rejection can still occur (acute or chronic).<br><br>Infection<br>Anti-rejection drugs suppress the immune system, increasing the risk of infections, including:<br>Common bacterial/viral infections

Prompt	NKF	ChatGPT
	many people will need more than one kidney transplant during a lifetime.	Opportunistic infections (e.g., CMV, fungal infections)<br><br>Medication Side Effects<br>Immunosuppressants can cause:<br>High blood pressure<br>Diabetes<br>High cholesterol<br>Weight gain<br>Bone thinning<br>Increased risk of certain cancers (especially skin cancer and lymphoma)<br>Surgical Complications<br>Bleeding, blood clots<br>Leakage or blockage of the ureter<br>Delayed function of the kidney (the kidney might not work immediately)<br><br>Long-term Risks<br>Chronic Rejection<br>Gradual loss of kidney function over time, even with treatment.<br>Cancer<br>Increased risk due to prolonged immune suppression.<br>Cardiovascular Disease<br>Transplant recipients are at higher risk of heart disease and stroke.<br><br>Psychological and Emotional Risks

Prompt	NKF	ChatGPT
		Anxiety about the transplant outcome<br>Stress from lifestyle changes and long-term medication use<br>Guilt (especially if the kidney came from a living donor)<br><br>Why It’s Still Often Worth It<br>Despite the risks, kidney transplants generally offer:<br>Better quality of life than dialysis<br>Longer life expectancy<br>Greater dietary and lifestyle freedom
4. Who can get a kidney transplant	In general, kidney patients of all ages can be considered for transplant. Some transplant centers have an upper age limit, while others don’t have any age limit. Every person will complete a full medical and psychosocial evaluation at a transplant center to make sure they are a candidate for transplant. Candidates for transplant need to be healthy enough for the surgery and prepared to take care of the new kidney, including taking medications every day. In many cases, people who are older or have other health conditions like diabetes can still have successful kidney transplants.<br><br>Remember – being put on the waitlist is not automatic. If you are interested in getting a kidney transplant, you must go to a transplant center for an evaluation.	Anyone with severe kidney disease or kidney failure (usually end-stage renal disease or ESRD) may be eligible for a kidney transplant, but several factors determine who can actually receive one. Here's a breakdown:<br>Who Can Get a Kidney Transplant<br>You may be eligible if:<br>You have end-stage kidney disease (your kidneys are functioning at <10–15%)<br>You're on dialysis or close to needing it<br>You're in generally good overall health (excluding kidney function)<br>You're able to follow a post-transplant care plan (medications, appointments, lifestyle)<br>You don’t have certain active infections or cancers<br>You don’t have severe heart, lung, or liver issues that would make surgery risky

Prompt	NKF	ChatGPT
		Who Might Not Be Eligible (or Need Further Evaluation)<br>People with untreated or ongoing cancer<br>Those with severe heart disease or other major medical conditions<br>Individuals with active infections<br>People who can’t follow a strict treatment regimen (e.g., due to cognitive issues or substance use without support)<br>People with a short life expectancy due to other health issues<br><br>Other Considerations<br>Age isn't a strict limit — both children and older adults can receive transplants, depending on health.<br>Living donors (usually family or friends) can sometimes speed up the process.<br>If a compatible kidney isn’t available, people can be added to the transplant waiting list.

Table S2. Comparison of GPT-4o and GPT-5.1 Responses

**Source**	**Word Count (Mean ± SD)**	**Flesch–Kincaid (FK) Grade Level (Mean ± SD)**	**Wilcoxon Test**
GPT-4o	219 ± 18.62	17.12 ± 6.83	Word Count: V = 0, p = 0.13<br>FK grade: V = 8, p = 0.38
GPT-5.1	302 ± 59.64	14.06 ± 2.36	

Note. Flesch–Kincaid (FK) grade level was calculated to assess the reading difficulty of each response. Due to the small number of paired items (n = 4 FAQs), differences between GPT-4o and GPT-5.1 were evaluated using the Wilcoxon signed-rank exact test, a non-parametric test that does not assume normality. No statistically significant differences were observed for word count or FK grade level between the two GPT versions.
